# Insight in Hypoxia-Mimetic Agents as Potential Tools for Mesenchymal Stem Cell Priming in Regenerative Medicine

**DOI:** 10.1155/2022/8775591

**Published:** 2022-03-26

**Authors:** Agata Nowak-Stępniowska, Paulina Natalia Osuchowska, Henryk Fiedorowicz, Elżbieta Anna Trafny

**Affiliations:** ^1^Biomedical Engineering Centre, Institute of Optoelectronics, Military University of Technology, Warsaw, Poland; ^2^Laser Technology Division, Institute of Optoelectronics, Military University of Technology, Warsaw, Poland

## Abstract

Hypoxia-mimetic agents are new potential tools in MSC priming instead of hypoxia incubators or chambers. Several pharmaceutical/chemical hypoxia-mimetic agents can be used to induce hypoxia in the tissues: deferoxamine (DFO), dimethyloxaloylglycine (DMOG), 2,4-dinitrophenol (DNP), cobalt chloride (CoCl_2_), and isoflurane (ISO). Hypoxia-mimetic agents can increase cell proliferation, preserve or enhance differentiation potential, increase migration potential, and induce neovascularization in a concentration- and stem cell source-dependent manner. Moreover, hypoxia-mimetic agents may increase HIF-1*α*, changing the metabolism and enhancing glycolysis like hypoxia. So, there is clear evidence that treatment with hypoxia-mimetic agents is beneficial in regenerative medicine, preserving stem cell capacities. These agents are not studied so wildly as hypoxia but, considering the low cost and ease of use, are believed to find application as pretreatment of many diseases such as ischemic heart disease and myocardial fibrosis and promote cardiac and cartilage regeneration. The knowledge of MSC priming is critical in evaluating safety procedures and use in clinics. In this review, similarities and differences between hypoxia and hypoxia-mimetic agents in terms of their therapeutic efficiency are considered in detail. The advantages, challenges, and future perspectives in MSC priming with hypoxia mimetic agents are also discussed.

## 1. Introduction

The proper functioning of human tissues and organs depends on natural regeneration processes. The regenerative potential is primarily maintained by the stem and progenitor cells whose progeny replace aged or injured cells when needed [1–3]. Mesenchymal stem cells (MSCs) are stromal cells that self-renew and display multipotency, together with unique immunomodulatory properties. Numerous studies are currently carried out on MSCs to treat neurodegenerative or immune-derived inflammatory diseases [1, 4, 5]. MSCs can be isolated from adult tissues (e.g., bone marrow (BM), adipose tissue (AD), skeletal muscle (SM), and dental pulp (DP)) [6–9] or fetal tissues (e.g., placenta, amniotic fluid (AF), Wharton jelly (WJ), and umbilical cord (UC)) [10, 11]. Epidermal stem cells, multipotent skin-derived precursors, and other stem cells can also be efficiently isolated from human skin [12].

By July 2020, 1,138 clinical trials have been registered at clinicaltrials.gov [13], mostly in traumatology, pneumology, neurology, cardiology, and immunology [14–19]. Most registered cases were in phases 1 (Ph1) and 2 (Ph2) of clinical trials. The percentage of particular phases of clinical trials in the fields mentioned above is as follows: in traumatology (total 234 cases), 30.7% in Ph1, 58.5% in Ph2, 9.8% in Ph3, and 0.8% in Ph4; in pneumatology (total 99 cases), 43.4% in Ph1, 53.5% in Ph2, 3.0% in Ph3, and 0% in Ph4; in neurology (total 97 cases), 31.9% in Ph1, 62.8% in Ph2, 4.1% in Ph3, and 1.0% in Ph4; in cardiology (total 83 cases), 25.5% in Ph1, 60.2% in Ph2, 14.4% in Ph3, and 0% in Ph4; and in immunology (total 78 cases), 17.9% in Ph1, 64.1% in Ph2, 17.9% in Ph3, and 0% in Ph4 [13, 16]. The outcomes of 18 clinical tests have already been described [16], and bone marrow was the most common source of isolated cells. MSCs have found potential applications in the treatment of multiple sclerosis (MS), Crohn's disease (CD), diabetes mellitus (DM), graft-versus-host disease (GVHD), rejection after liver transplant, liver disorders [5], and acute and chronic wounds [20, 21].

Despite such notable progress, there are still numerous challenges. Clinical applications demand systemic administration of the high number of stem cells (50–200 million per patient) [16, 22]. The number of stem cells in human tissues is usually small [23], and their efficient proliferation *in vitro* is challenging [24–26]. Both MSC aging and spontaneous differentiation are factors that may occur *in vitro*. The isolated stem cells are usually grown *in vitro* under ambient conditions where oxygen concentration is four to ten times higher than in a stem cell niche [27–30]. Thus, high oxygen concentration upon MSC culture results in early senescence and nuclear damage and may increase the doubling time [31–33]. Poor MSC engraftment after transplantation was also revealed [34].

Over the last few years, numerous low oxygen priming approaches have been explored for MSC clinical application [35, 36]. MSCs growing under hypoxia [37] demonstrate enhanced proliferation, immunomodulatory properties [38–43], efficient survival, and neovascularization after grafting.

As such, several hypoxia-mimetic agents can be used to induce hypoxia in tissues, e.g., deferoxamine (DFO), dimethyloxaloylglycine (DMOG), 2,4-dinitrophenol (DNP), cobalt chloride (CoCl_2_), and isoflurane (ISO). Could they effectively replace the hypoxia chambers/incubators in the MSC priming? This review looks for an answer to this question and discusses similarities and differences between the effects of hypoxia and hypoxia-mimetic agents. The oxygen concentration, incubation time, and MSC therapeutic proficiency are described in detail. Since there are still some ambiguities in the literature regarding hypoxia as a standard approach in MSC production, this issue will be extensively discussed.

To summarize the recent findings on hypoxia in this review, we searched PubMed, Scopus, Science Direct, and Web of Science databases from 2006 to September 2021 for potentially relevant studies published in English. Original papers, systematic reviews, and book chapters were reviewed. The search strategy first has focused on critical terms: hypoxia, hypoxia mimetic agents, mesenchymal stem cells, and clinical applications of MSC. These criteria have been extended with the more detailed terms: application in regenerative medicine, cell treatment, cell-based therapies, mesenchymal cells' source (Warton jelly, umbilical cord, bone marrow, umbilical cord blood, adipose, and dental pulp originated from human, rat, and mouse), and chemicals: deferoxamine, cobalt chloride, isoflurane, dimethyloxaloylglycine, and 2,4-dinitrophenol. We excluded studies enrolling hypoxia/hypoxia-mimetic agents together with specific adjuvants such as immunomodulators.

## 2. Role of Hypoxia in a Stem Cell Niche

The stem cell niche is a microenvironment, which governs stem cell's functions and fate [44]. Morphogens, growth factors, cytokines, oxygen tension, extracellular matrix, and shear stress could affect stem cells within the niche [5, 45].

MSCs can be found in the niches close to blood capillaries throughout the body [46]. The oxygen concentration in the tissues where MSCs reside is low despite their efficient vascularization [47, 48]. The oxygen concentration is much lower in human tissues than in inhaled air (21%). It happens because the oxygen concentration of the inhaled air constantly drops, entering the lungs, and when it reaches organs and tissues, its concentration ranges from 2% to 9% [49, 50]. Since the concentration of O_2_ in blastocysts and stem cells niches is very low, oxygen tensions tend to be critical in their metabolic milieu. Hypoxia sustains the phenotype of hematopoietic, embryonic, neural, and mesenchymal stem cells and influences stem cells' function and fate. Furthermore, hypoxia acts on stem cells via different molecular pathways, including signaling of homolog translocation-associated (Drosophila) (Notch) and octamer-binding transcription factor 4 (Oct4), the stemness controllers [25].

Stem cells are physiologically adapted to hypoxia. Therefore, hypoxic priming should maintain MSCs in an undifferentiated state and preserve their functions and plasticity.

## 3. Hypoxia versus Hypoxia-Mimetic Agents for MSC Priming

Injured tissues have poor vascularization (especially in ischemic injuries) and cannot maintain the metabolism of implanted not-primed MSC at an appropriate rate; therefore, most cells undergo apoptosis soon after transplantation. It is due to stem cells grown in normoxia not adapting quickly to the conditions of hypoxia. Hence, to survive after transplantation, stem cells must be trained ex vivo to sustain hypoxia conditions [51].

The simplest solution is to cultivate MSCs under low oxygen conditions. Various hypoxia incubators and chambers were used for MSC culture. However, both have limitations in their use [52]. They suggest that pharmaceutical/chemical agents are more valuable because they provide higher oxygen tension stability than hypoxic chambers and are not expensive [53].

Now the question arises whether pharmacological or chemical hypoxia-mimetic agents act similarly on stem cells. Before answering this question, we intend to discuss the influence of hypoxia on the crucial MSC features.

### 3.1. Cell Surface Markers and Morphology

The most important MSC feature is their immunophenotype that defines their stemness according to the International Society for Cellular Therapy (ISCT). MSCs express CD90, CD105, and CD73 antigens and do not express CD11b, CD14, CD19, CD45, CD34, and CD79a antigens, nor human leukocyte antigen-DR isotype (HLA-DR). The proteins SRY-box transcription factor 2 (SOX2) and Oct4 occur in embryonic stem cell- (ESC-) like [54]. The expression of other surface markers depends on the MSC tissue source. Homeobox transcription factor NANOG, reduced expression-1 (REX-1), T cell receptor alpha locus 1-60 (TRA-1-60), TRA-1-81, stage-specific mouse embryonic antigen (SSEA-3), and SSEA-4 markers have been found on MSCs isolated from human liver and fetal blood but not on the cells derived from adult bone marrow [55, 56].

The influence of the hypoxia priming on MSC's surface markers is summarized in Table 1. Hypoxic conditions in the oxygen range of 2-5% preserve the expression of surface markers on MSCs. Only in low oxygen concentration of 1% are the results inconclusive. The expression of negative surface markers is maintained at 1% O_2_ [57–59], but some studies showed a reduced expression of positive markers. Compared to normoxia, CD44 and CD105 reduction on the MSC surface from 90% to 75% and from 99.4% to 94.9%, respectively, was noted [57]. Upregulation of other stem cell markers as Oct4, REX-2, or NANOG was presented [38, 60].

Of no less importance is maintaining the appropriate morphology of MSCs growing in confluence. While increased cellular density and number of passages significantly change MSC's morphology under normoxia and cause cell retraction at high density, hypoxic conditions retain the MSC's spindle shape, and cells can divide even at high density, permitting multilayer formation [38]. Similarly, MSCs treated with a hypoxia-mimicking agent DFO did not alter their morphology. However, some intracellular vacuole-like structures may occur within the cells [61].

To summarize, the expression of stem cell surface markers is generally preserved under hypoxia but depends on the oxygen concentration, exposure time, tissue, and donor of MSCs. Up to date, there are no data on the influence of hypoxia-mimicking agents on MSC surface markers expressions.

### 3.2. Viability, Proliferation, and Clonogenicity

A high proliferation rate is critical for the successful implementation of stem cell-based therapy. The oxygen concentration and the incubation time may influence the overall hypoxia effect on stem cells, especially their viability, proliferation, and clonogenicity.

#### 3.2.1. Hypoxia

As shown in Table 2, the proliferation, viability, and clonogenicity of the stem cells derived from various tissues were studied under at least 30 conditions different in terms of oxygen concentration and incubation time under reduced oxygen concentration.

In 19 conditions, an increase in proliferation or clonogenicity of MSCs was observed in the oxygen concentration ranged from 1 to 5%. Out of these 30 conditions considered, a decrease in cell viability was recorded in eight. This discrepancy is not related to oxygen concentrations since proliferation inhibition was observed at both 1% and 5% oxygen concentrations. It also does not depend on the time of cell growth under hypoxia because inhibition of proliferation was observed both after 2-day exposure to reduced oxygen concentration and after 21-day exposure at similar oxygen concentrations. These divergent effects can also be seen on one type of stem cell, e.g., BM-MSCs. Likewise, the impact of the test method on the results obtained cannot be attributed, e.g., Trypan Blue staining and counting cells under the microscope were used at the elaboration of conditions resulting in discrepant observations. It is, therefore, possible that more subtle molecular phenomena occurring in stem cells while growing under hypoxic conditions should be investigated, such as transcriptome or metabolome of hypoxia-treated cells.

The higher proliferation of MSCs could be attributed to the transition from aerobic to anaerobic respiration through oxidative phosphorylation and glycolysis, respectively [67]. The increase in glucose consumption and lactate generation in UC-MSCs in hypoxic culture may exemplify the metabolic changes described above and require enhanced glucose transport into the cells. Increased MSC proliferation under hypoxia enhances glucose uptake as the critical carbon source for the biosynthesis of essential nutrients. The involvement of metabolic pathways as glycolysis (lactate dehydrogenase A, LDHA), oxidative phosphorylation (3-phosphoinositide-dependent protein kinase 1, PDK-1), cellular glucose transport (cellular glucose transporter-1, GLUT-1), pentose phosphate pathway (glucose-6-phosphate dehydrogenase, G6PD), and the significant targets of hypoxia-inducible factor 1 alpha (HIF-1*α*) transcriptional factors was demonstrated in the diminished oxygen concentration [63, 68, 69].

HIF-1*α* is a crucial transcriptional factor, which regulates the adaptive response to hypoxia. Many proteins react directly with HIF-1*α* enhancing or reducing their activities. HIF-1*α* stabilization improves MSC's proliferation rate and may augment their therapeutic potential [70].

The reduction of cellular senescence and inhibition of the telomere shortening was also observed in MSCs under hypoxic conditions [62, 63, 71, 72]. The mechanism of apoptosis suppression under hypoxia might relate to the cellular tumor antigen (p53) pathway inhibition [73]. The decreased O_2_ tension could also lead to the lower level of reactive oxygen species (ROS); the primary factor was attributed to increased cellular damage [74].

*(1) MSC's Gene Expression*. Cells' adaptation to hypoxia requires changes in molecular pathways. Hypoxia regulates the transcription of hundreds of genes, which play a role in oxygen-dependent functions like angiogenesis, glycolysis, metabolism, proliferation, and apoptosis [75]. Most of these changes are HIF-1*α*-dependent and transcriptionally regulated. HIF-1*α* is also subject to epigenetic mechanisms such as histone modification, DNA methylation, and noncoding RNA-associated gene silencing [76]. Thus, epigenetic modifications are additional mechanisms regulating gene expression in hypoxia and enhancing or inhibiting their activity. However, the contribution of microRNA (miRNA) functioning during hypoxia and DNA methylation is not yet fully understood [77]. Furthermore, molecules of short noncoding RNAs and miRNAs, which regulate gene expression, are controlled by hypoxia in stem cell niches [78]. Some miRNA regulate vascular endothelial growth factor (VEGF), which stimulates angiogenesis and tightly controls hypoxia-induced cellular alteration [77, 79].

Beyond epigenetic mechanisms, hypoxia upregulates over 135 genes governing several physiological pathways, e.g., glycolysis, metabolism, proliferation/survival, transduction, and signaling transduction in BM/umbilical cord blood- (UCB-) MSCs in the oxygen range from 1.3% to 10% [75, 82]. Short-term hypoxia downregulates proapoptotic genes such as BCL-2-associated X (*BAX*), B-cell lymphoma 2 (*BCL-2*), and caspase 3 (*CASP-3*) (Table 2), thus preventing cells from cellular damage after transplantation [57].

Hypoxic conditions (1-5% O_2_) increased expression of *HIF-1α* in BM-, UCB-, umbilical cord (UC), and WJ-MSCs [58, 64, 69]. Only Antebi et al. noted a downregulation of *HIF-1α* under 1% hypoxia in BM-MSCs [57]. Upregulation of energy metabolism-related genes *GLUT-1*, *PDK-1*, and *LDH* was noticed in 1.5, 2.5, and 5% O_2_ [69]. Overexpression of *Slc16a3* (a gene of monocarboxylate transporter-4, MCT-4) under prolonged hypoxia in mBM-MSC was noted [68]. The expression of the proliferative/survival genes *Vegf-d*, placental growth factor (*Pgf*), and matrix metalloproteinase 9 (*MMP-9)* was also elevated in 1.3 and 10% O_2_ compared to normoxia [75]. The rise in *Notch*, Notch ligand, and *JAGGED* was observed, suggesting a link between hypoxia and Notch signaling pathway. Moreover, the augmented proliferation of hWJ-MSCs under hypoxia confirms the Notch-related proliferation [64].

*(2) Reoxygenation of MSCs in Culture*. As mentioned in Table 2, there is another way to grow cells with limited oxygen availability. It includes 15 min of preconditioning at 2.5% O_2_, 30 min of reoxygenation in ambient conditions, and the final conditioning at 2.5% O_2_ for 72 h. Such conditions were used for hUB-MSC culture, significantly improving the cell proliferation and migration *in vitro*.

The reoxygenation process following short hypoxia priming enhanced the prosurvival genes' expression together with numerous angiogenic and trophic factors, such as the basic fibroblast growth factor (bFGF) and VEFG in MSCs [18, 60, 84, 86]. Moreover, other positive effects include the reduced release of lactate dehydrogenase, lower activity of apoptosis-related caspases, and diminished cell sensitivity to ischemia resulting from the reoxygenation of the MSC culture [57, 87].

*(3) Spheroids*. Using a spheroid with short-term hypoxia in 1% O_2_ poses an advantage over transplantation of individual cells. Spheroids better mimic cellular behavior in native tissue, improving viability, angiogenesis, and immunomodulatory properties [88]. Moreover, interactions of MSCs with endogenous ECM within spheroids increase proliferation and maintain osteogenic differentiation potential influencing bone tissue repair. The synergy of MSC priming with hypoxia and MSC spheroid transplantation is believed to be a good cellular therapy due to increased survival, angiogenic potential, and bone formation. Moreover, spheroids enhance interaction with ECM and promote osteogenesis. Thus, MSC priming under hypoxia and spheroids grafting can be effective in regenerative medicine [89].

#### 3.2.2. Pharmacological and Chemical Hypoxia-Mimetic Agents

Among commercially available pharmaceutical/chemical hypoxia-mimetic agents, the following are discussed below: DFO, DMOG, DNP, CoCl_2_, and ISO.

DFO is a chelating agent used to remove an excess of iron or aluminum from the body [90]. DFO stabilizes HIF-1*α* under normoxia; thus, it is a suitable hypoxia-mimetic agent [91]. DMOG is a prolyl hydroxylase inhibitor. DMOG regulates HIF-1*α* and phosphorylation under hypoxia. DMOG acts via inhibition of factor inhibiting HIF-1*α* (FIH-1) and the prolyl hydroxylases via competitive inhibition of 2-oxoglutarate (2-OG). It indicates that DMOG can be an effective drug for diabetes due to HIF-1*α* regulation [92, 93]. DNP increases oxygen consumption due to the enhancement of oxidative metabolism [94]. CoCl_2_ artificially induces hypoxia and can block the degradation of HIF-1*α* protein, thus inducing its accumulation [52, 95–97]. ISO is a volatile anesthetic agent. Because of its cytoprotective capacities, it is a good candidate to be a hypoxia-mimetic agent that activates HIF-1*α* [98].

*(1) Cytotoxicity*.  Table 3 presents the results on the MSC viability upon pharmacologically- or chemically induced hypoxia.

Most studies have been carried out with DFO. It was used in a concentration range of 0.1-500 *μ*m. DFO did not impair the viability of MSCs until 120 *μ*M [97, 99, 100]. The standard preconditioning protocol of MSC treatment with DFO (48 h at a concentration of 3 *μ*M) can be substituted with treatment for 12 hours at a concentration of 50 *μ*M [99]. Fujisawa et al. showed significant cytotoxicity of DFO at a concentration of 10 *μ*M towards BM-MSCs but only after long-term treatment of 53 days [61].

The viability of BM-, UC-, AD-, and DP-MSCs was preserved when CoCl_2_ was used for 24-48 hours at a concentration of 100 *μ*M [101]. CoCl_2_ at a concentration of 500 *μ*M significantly decreased MSC viability [102]. DMOG is noncytotoxic until it reaches a concentration of 5 mM [103]. DMOG also increased the proliferation of cocultured cell BM-MSC and human umbilical vein endothelial cells (HUVEC) [52]. ISO increased hBM-MSC metabolism at a concentration of 2% and incubation time of 4 h [98]. DNP at a concentration of 0.25 mM did not injure rBM-MSCs in the coculture with cardiomyocytes, but the treatment period was very short (20 min). Otherwise, this compound could be highly toxic. The cells were slightly shrunken but regained normal morphology after their reoxidation for 2-24 hours. Thus, these results imply that the differences in culture protocols and compound concentrations may be crucial for successfully implementing hypoxia and hypoxia-mimetic agents in regenerative medicine.

*(2) Metabolome*. The metabolic changes occur in the cells upon adaptation to hypoxia. Metabolome analysis revealed that both hypoxia treatment and DFO administration influence cellular metabolism.

MSCs exhibited metabolic changes in Krebs tricarboxylic acid (TCA) cycle, amino acids, creatine, uric acid, and purine and pyrimidine metabolism upon both types of treatment. DFO-derived hypoxia affected TCA cycle-related metabolism by increasing aconitate, alpha-ketoglutarate (*α*-KG), and citrate concentrations and decreasing malate and fumarate via reductive carboxylation in reverse Krebs cycle. These effects were more visible for DFO-induced than natural hypoxia (increase only in the *α*-KG level) [61]. *α*-KG provides energy for the cellular oxidation of nutrients. The increased *α*-KG level is required during enhanced cell proliferation. As a precursor of glutamate and glutamine, *α*-KG acts as an antioxidant agent and directly reacts with hydrogen peroxide. DFO stronger upregulated *α-KG* in comparison to hypoxia, providing better protection against ROS [104].

The low level of malate and fumarate during hypoxia had a positive effect on cells. In contrast, high levels of these compounds were harmful and led to cancer development (by mediating chronic proliferative signals) [105, 106].

The impairment of purine and pyrimidine metabolism is also detrimental to cells, and elevated uric acid levels generated from the purines' metabolism may be responsible for human diseases such as vascular inflammation, atherosclerosis, articular, and gout degenerative disorders [107]. Since phosphoribosyl pyrophosphate (PRPP) is an enzyme involved in synthesizing purine and pyrimidine nucleotides, its level raised under DFO-derived hypoxia [61, 108].

Additionally, 1% hypoxia upsurges the level of the 1-methyl adenosine, a stress marker, compared to DFO-primed MSCs [61, 109]. Further detailed investigations on this topic are required [61, 110, 111].

To summarize, DFO-induced hypoxia affects minor MSC metabolic changes compared to hypoxia. Up to now, detailed metabolome studies have been done only for DFO. Metabolome studies of other hypoxia-mimetic agents are needed to understand the mechanism of their actions and possible short- and long-term side effects.

*(3) MSC's Gene Expression*. All hypoxia-mimetic agents discussed here increase the expression of HIF-1*α*, the central controller of adaptive cellular response to hypoxia, and enhance glycolysis similarly to hypoxia [4, 53, 61, 98, 99]. DFO upregulates the genes related to glycolysis: hexokinase 2 (*HK2*), *PDK-1*, BCL-2 interacting protein 3 (*BNIP3*), and *LDHA* [61]. DFO upregulates *NUPR* and *p16* expression, improving cell survival [99]. It also induces an increase in the level of HIF-1*α* by 50-110% while DMOG elevates HIF-1*α* level by 2-3 times, which is less than CoCl_2_ stimulating HIF-1*α* by 2-5 times compared to normoxia. ISO demonstrated the highest impact on the HIF-1*α* expression (a 150-400% increase). Moreover, DMOG via increasing of HIF-1*α* expression and activation of the phosphoinositide 3-kinases/protein kinase (PI3K/Akt) signaling pathways regulates cell survival and apoptosis [103]. DMOG lowers myocardial apoptosis [112] via the PI3K/Akt pathway activation. Stabilization of HIF-1*α* and activation of the PI3K/Akt pathway are crucial for VEGF upregulation.

### 3.3. Differentiation

This subchapter presents the effects of hypoxia and pharmaceutical/chemical hypoxia-mimetic factors on MSC differentiation. The ability to the multidirectional differentiation is a crucial hallmark of MSC. Furthermore, the differentiation potential and proliferation rate of MSC depend on the type of cells source.

#### 3.3.1. Hypoxia

As described above, stem cells adapt metabolically to hypoxia *in vitro* [113]. The question is whether they differentiate equally efficiently in hypoxia compared to normoxia. The cells can be grown under hypoxia before induction of the differentiation process by the appropriate media (a pretreatment), or lower oxygen tension may be maintained in cultures during differentiation (a treatment). In Table 4, we summarize the available data on the influence of hypoxia on the fate of MSCs cultured in the growth or differentiation media.

Hypoxia pretreatment and treatment can maintain or reduce MSC's osteogenic potential. These effects were observed at the oxygen concentration ranging from 1 to 5% for BM-, AD-, UCB-, UC-, and WJ-MSCs. It may be related to the low expression of the *ALP* and *ALPL* genes coding for *alkaline phosphatases* and the *IBSP* gene coding for an integrin-binding sialoprotein in AD- and BM-MSCs. However, Boyette et al. noted increased *BGLAP*, *RUNX2*, and *COLL2* in hBM-MSC [65].

Hypoxia pretreatment and differentiation in low oxygen conditions (1-5% O_2_) preserve BM-, AD-, UCB-, UC-, and WJ-MSC capability for adipogenic differentiation [84, 114]. In BM-, AD-, UCB-, UC-, and WJ-MSCs, the expression of the following adipogenic marker genes, lipoprotein lipase (*LPL*), *PPARα*, peroxisome proliferator-activated receptors (*PPARγ*), and fatty acid-binding protein 4 (*FABP4*), was preserved or even increased.

Nevertheless, inconclusive observations concern the ability to differentiate into cartilage. Chondrogenic potential might be elevated under hypoxia pretreatment [62, 81, 82, 84, 116] and maintained or reduced during hypoxic differentiation in WJ- and BM-MSC in 1-2% O_2_ [64, 65]. The expression of chondrogenic marker genes SRY-box transcription factor 9 (*SOX9*) and collagen type II alpha 1 chain (*COL2A1*) followed the above pattern in AD-, BM-, and UCB-MSCs.

Hypoxia pretreatment/treatment influences the MSC differentiation process with effectivity related to passage numbers. In primary cell lines and at the low number of passages, MSCs maintain their differentiation potential compared to the cells passaged many times in the *in vitro* culture under hypoxia [31, 66, 116]. The downregulation of the *FABP4*, *LPL*, *ALPL*, and *IBSP* genes accompanied this diminished capacity of MSCs.

Moreover, individual stem cells under hypoxia are characterized by the enhanced level of plasticity-dependent marker genes such as *NANOG*, *REX-1*, or *Oct4* [117]. The increase in osteogenic potential of individual MSCs was observed compared to monolayer cell culture under normoxia [118]. Oct4 is an essential transcription factor for self-renewal, and it is present in MSCs at low levels on each passage (the higher passage number, the lower Oct4 level). Improved stemness due to higher expression of *Oct4* can result in increased differentiation potential of hypoxia primed stem cells [89, 119].

#### 3.3.2. Pharmacological and Chemical Hypoxia-Mimetic Agents

According to Table 5, the DFO-derived hypoxia treatment during differentiation preserves osteogenic potential and the level of its corresponding marker genes *ALP* and Runt-related transcription factor 2 (*RUNX2*).

DFO treatment maintains or reduces adipogenic potential while increasing chondrogenesis and the expression of *SOX9*. These effects were observed in BM- and UC-MSC after 14-21 days of treatment [61, 97, 120, 121].

CoCl_2_-derived hypoxia pretreatment increased osteogenesis and upregulated the *Alp*, *Col1*, and osteocalcin (*Bglap*) genes while treatment during differentiation maintained osteogenic potential and the expression of *RUNX2*, *ALP*, and *COLLI.* These effects were observed on mC3H/10T1/2 MSCs and UC-MSCs for 1-9 days [52, 97, 101]. Murine C3H10T1/2 cells are embryogenic cells with features of mesenchymal stem cells and thus represent interesting research objects. They have the potential to be an attractive alternative source of primary BM-MSCs in studies of osteogenic and chondrogenic differentiation for regenerative medicine [122]. CoCl_2_-derived hypoxia pretreatment decreases adipogenesis and the marker genes Apetala 2 (*aP2*), CCAAT/enhancer-binding protein *α* (*C/ebpα*), and *Pparγ* in mC3H/10T1/2 MSC for 24-48 hours. On the opposite, CoCl_2_-derived hypoxia treatment maintained adipogenicity in BM-MSCs for eight days [101, 123]. CoCl_2_-derived hypoxia pretreatment and treatment increase chondrogenesis and the expression of the chondrogenic marker genes *SOX9*, *Coll2a1*, *VCAN*, and aggrecan (*ACAN*) in mC3H/10T1/2, BM-, UC-, AD-, and DP-MSCs for 2-21 days [53, 101, 124].

DMOG-derived hypoxia treatment of BM-MSCs maintained osteogenesis and *RUNX2* expression and upregulated *ALP* and *COLLIA1* for nine days [52] as well as chondrogenesis and *SOX9* marker for 21 days [124].

The bone fracture niche is hypoxic; therefore, oxygen tension is critical in bone healing. Nguyen et al. performed a direct coculture of BM-MSCs and HUVEC in normoxia and the chemically activated hypoxia with CoCl_2_ and DMOG. Under hypoxia induced by CoCl_2_, von Hippel–Lindau protein (pVHL) which binds to the oxygen-degrading domain and prevents hydroxylation of HIF-1*α* by oxygen-dependent prolyl hydroxylases (PHD) was inhibited [127]. DMOG can directly inhibit PHD and stabilize HIF-1*α*[124, 128] compared to normoxia. Normoxia generally promotes bone formation in MSCs and HUVEC coculture, while hypoxia favors angiogenesis. DMOG is a more promising hypoxia-priming agent than CoCl_2_ because it stronger enhances endothelial marker—von Willebrand factor (VWF) and VEGF [129]. Moreover, coculture (in the ratio 1 : 1) of BM-MSCs and HUVEC promotes osteogenesis in MSCs under normoxia, and hypoxia even enhances this effect [52].

Unfortunately, up to date, no data on the influence of DNP or ISO on MSC differentiation are available in the literature. Concerning the published results, it may be assumed that the effect of pharmaceutical/chemical hypoxia-mimetic agents on MSC differentiation is similar to hypoxia. However, the period of this enhancement has not been studied yet. The currently available scientific data also do not allow concluding whether hypoxia-inducing chemical agents could efficiently reduce the time required for cell differentiation.

### 3.4. Engraftment, Migration, and Secretion Profile

Successful MSC engraftment is crucial in regenerative medicine. The high proliferation rate and prominent expression of chemokine receptors on MSCs are attributed to young cells providing migration and potential therapeutic increase after transplantation [45].

The latest data indicate that chemokines and their receptors are critical in migration, chemotaxis, and homing *in vitro* and *in vivo* [130]. There are different BM-MSC-related chemokine receptors, such as CXC, but insufficient data are available on their function in cell therapy [131]. The rat brain ischemia model shows that chemokines C–C motif *chemokine* ligand 2 (CCL25) and C-X3-C motif chemokine ligand 1 (CX3CL1) can also influence MSC chemotaxis [131]. Moreover, CC-type chemokines are involved in cellular implantation and remodeling following transplantation [130].

HIF-1*α* causes upregulation of chemokine receptors on MSCs [132]. Under hypoxic conditions, the stabilized HIF-1*α* is shifted into the nucleus to bind the HIF-1*β* forming heterodimer. Subsequently, the heterodimer attaches to hypoxia response elements (HREs) linked with CREB-binding *protein*/p300 protein (CBP/p300) [133, 134] and increases the expression of chemokine receptors C-X3-C motif chemokine receptor 1 (CX3CR1), C-X-C chemokine receptor type 7 (CXCR7), and C-X-C motif chemokine receptor 4 (CXCR4). Hypoxia can increase *CXCR4* expression [135]. Hypoxia-induced upregulation of *CXCR4* may result from HIF-1*α* stabilization [136]. Metabolic flexibility is one of the features represented by MSCs, helping them survive under ischemic stress and maintaining their multipotency [137]. HIF-1*α* is one of the master regulators controlling the cellular response to the tension caused by low oxygen levels [138].

HIF-1*α* is also involved in the CXCR4 expression induced by the activation of HREs in the Ets1 promoter, a transcription factor of CXCR4. Changes in the oxygen level are an essential regulator of CXCR4 expression. Hypoxia stabilizes CXCR4 transcripts, contributing to an increase in the *CXCR* gene expression. It suggests that hypoxia-regulated RNA binding proteins could influence CXCR4 stabilizing its mRNA at the posttranscriptional level [139].

Angiogenesis is vital in tissue engineering because of tissue blood flow restoration and new blood vessel formations [140]. Proangiogenic factors (VEGF and matrix metalloproteinases (MMPs)) and antiangiogenic factors (endostatin and tissue inhibitor of metalloproteinases (TIMPs)) are involved in angiogenesis regulation [141]. Applications of proangiogenic proteins in stroke and myocardial infarction treatment have been reported [142].

#### 3.4.1. Hypoxia

As shown in Table 6, hypoxia increases MSC migration via upregulation of chemokine receptors *CXCR1* and *CXCR4*.

This effect was observed in the O_2_ concentration ranged from 1 to 5% when BM- and UC-MSC were grown for 8-48 hours [60, 81]. The *CXCR4* gene expression decreased in C57BL/6 murine BM-MSCs exposed to acute hypoxia compared to normoxia. The reduction of the *CXCR4 gene* expression could result from the long-term culture of cells in normoxia followed by acute hypoxia shock. In the next step, MSC reoxygenation after hypoxia led to the *CXCR4* gene expression decreasing. The reduction of the *CXCR4* gene expression during the second stage of reoxygenation could have been caused by the compatibility of cells to new oxygen conditions—hypoxia following the suppressive effect of normoxia on the *CXCR4* promoter [143].

Hypoxia also increases the angiogenic capacity of MSCs. This effect might be observed upon O_2_ concentration ranging from 1% to 5% after incubating BM-MSCs for 2-4 days [57, 80, 89]. The *VEGF* gene expression increased under hypoxic conditions [119, 144]. VEGF and Angiopoietin 1 (Ang-1) play a crucial function in angiogenesis, and their increase is essential for successful stem cell transplantation [102, 145]. Decrease of high mobility group box protein 1 (HMGB1) nuclear protein under hypoxia is believed to protect tissue from damage [80]. MSC's spheroids promote vascularization and bone formation [89].

Cell migration, vascularization, and tissue remodeling in bone are MMP/TIMP dependent. The family of TIMP proteins controls MMPs' function. MMP-2, MMP-9, MMP-13, and TIMP-1 are crucial in bone formation and repair [65]. MMP-9 and MMP-13 are involved in the recruitment and activation of osteoclasts [146–148]. MMP-2 is essential for generating spatial osteolytic structures and mineralization [149]. A loss of its function can disrupt proliferation and osteoblastic differentiation, disturbing skeletal development [150], and mutation in the *MMP-2* gene might cause bone diseases [151]. Hypoxic preconditioning showed upregulation of many *MMP* and *TIMP* genes in 5% O_2_ up to ten days in BM-MSCs [65, 152]. Long-term hypoxic cultivation upregulates *MMP7-16* and *TIMP1-3* but downregulates *MMP2*. There are few experiments on this topic, but it requires further investigation.

Heart damage is one of the common diseases of modern civilization [153]. Cardiomyocytes, endothelial cells, fibroblasts, and perivascular cells are crucial in heart homeostasis. Transplantations of two cell types, cardiomyocytes (CMs) and vascular cells, exhibited better therapeutic effects in infarcted hearts [154]. Moreover, the coculture of myocytes with endothelial cells enhances myocytes' survival *in vitro*[155]. New, more efficient strategies are still needed. Mathieu et al. noted that hESC could reenter pluripotency under hypoxia conditions, and this dedifferentiation depends on HDAC activity [156]. The iPSC research seems to be very promising, as it does not raise ethical questions such as the hESC [157]. Practical methods for differentiating murine iPSC-derived cardiomyocytes, combining hypoxia and bioreactor controlling culture conditions, have already been described [158]. Extracellular vesicles (EVs) are attracting the attention of researchers because of their ability to mimic all the therapeutic effects induced by the MSCs (e.g., anti-inflammatory, proangiogenic, or antifibrotic) [159]. Thus, MSC-derived EVs can modulate tissue response to a broad spectrum of injuries [160] and are considered a substitute for cell-based therapies. The clinical studies using exosomes in the treatment of cardiovascular disease are at an early stage [161–163]. For example, the exosomes derived from BM-MSCs [161, 163] or umbilical cord- (UC-) MSCs [164] showed the positive influenced cardiac function (preclinical model of MI) [165]. Hypoxia and DFO preconditioning of MSC for EV delivery is the developing strategy for regenerative medicine [166, 167].

#### 3.4.2. Pharmacological and Chemical Hypoxia-Mimetic Agents

As shown in Table 7, pharmaceutical/chemical hypoxia-mimetic agents can improve the migration and angiogenic capabilities of MSCs.

An increase in migration was observed after BM- and WJ-MSC incubation with DFO and ISO for 4-72 hours [98, 100, 115]. The increased expression of VEGF was noted in WJ-, AD, and BM-MSCs after their treatment with DFO, DMOG, or DNP for 20 min and 48 hours [94, 115, 168].

Preconditioning of MSC with DMOG was applied in the harvesting of cells for application in the treatment of heart ischemia [4, 92], cartilage regeneration [124], and bone regeneration in an aged population [45, 80]. DNP has already been used as a hypoxia-mimetic agent on numerous cell types such as neonatal cardiomyocytes, neurons, H9C2, and embryonic cardiac cells [169–171]. Preconditioning of stem cells with DNP improved their adhesion, survival, homing capacities, and cardiomyogenic genes such as *Gata*-binding *protein 4 (Gata-4)*, NKX2 homeobox *5 (Nkx2.5*), Connexin 43 (*Con43*), atrial natriuretic peptide (*Anp*), and *Vegf* [168]. MSC priming with DNP was used in the myocardium regeneration process [94] and improved cardiac function [168]. Similarly, preconditioning of MSCs with ISO improved their migration and engraftment into the ischemic brain (the rat model of stroke) [98].

Hypoxia increases migration and vascularization of MSCs and protects them against apoptosis. It was revealed that pharmaceutical/chemical hypoxia-mimetic agents stronger enhance the expression of chemokine receptors and VEGF than hypoxia. The exact effect depends on the hypoxia-mimicking agent. Moreover, chemokine receptor studies were performed only for DFO and ISO. There is no data about the influence of other hypoxia-derived agents on chemokine expression. Moreover, there was no information about essential proteins and MMP/TIMP changes upon treatment of MSCs with hypoxia-mimetic agents.

## 4. Conclusions

Clinical applications of MSCs gave insufficient effects due to low survival, retention, or the insufficiency of cell differentiation. Hypoxia conditions mimic the natural tissue environment preserving embryonic development and the pluripotency of stem cells and enhancing angiogenesis. The knowledge on MSC priming is critical in evaluating safety procedures and potential use in clinics. Hypoxia preconditioning *in vitro* uses 2-5% oxygen concentration. It preserves MSC's differentiation potential, upregulates chemokine receptors, and delays cell senescence in a source-dependent manner. There are clear pieces of evidence that both hypoxia pretreatment and treatment are beneficial for MSC differentiation. Hypoxia priming has been proved as a practical approach for ischemic stroke and other disability treatment.

**Figure 1:**
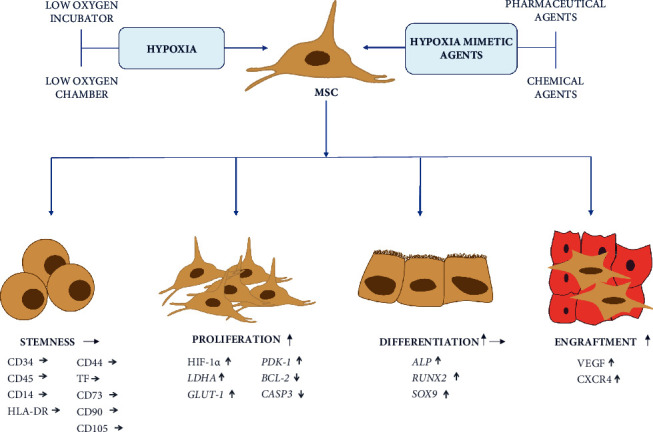
Hypoxic pretreatment methods for the stem cell efficient culture. Both hypoxia and hypoxia-mimetic agents can influence MSC homeostasis preserving their stemness. Only the major proteins and genes related are presented.

A growing group of pharmaceutical/chemical hypoxia-mimetic agents concur with hypoxia chambers and incubators, acting similarly according to the current knowledge (Figure 1). Pharmaceutical/chemical hypoxia-mimetic agents can also increase cell proliferation, preserve or enhance differentiation potential, increase migration potential, and induce neovascularization in a concentration- and stem cell source-dependent manner. According to the current knowledge, they act via upregulation of HIF-1*α*, leading to changes in the metabolism, e.g., increasing glycolysis. Pharmaceutical/chemical hypoxia-mimetic agents might find several applications in human medicine. DFO can be used in the general preconditioning of stem cells in regenerative medicine (due to contrary data on osteoblastic differentiation, its application in bone regeneration requires further investigation). CoCl_2_ is proposed for cartilage regeneration. DMOG has been applied in myocardial infarction, ischemic heart, brain, and bone regeneration in the aged population. Moreover, it is a better candidate for cartilage tissue regeneration compared to DFO and CoCl_2_. DNP is believed to promote cardiac regeneration, and ISO can be used in ischemic brain treatment.

However, current literature still shows certain contradictory data on the influence of hypoxia on MSC functions. This phenomenon stems from differences in the protocols used, culture conditions, media composition, hypoxia conditions and timing, and the heterogeneity of cell donors. At least on some hypoxia inducers, our knowledge of the mechanisms is not sufficiently comprehensive, affecting their potential use. Up to now, DFO is the most studied agent for MSC priming and seems to be a quite safe choice. Metabolome changes in DFO-derived hypoxia are less harmful to MSCs compared to hypoxia. Many new hypoxia-mimetic agents have not yet been fully characterized. One of these agents is DMOG, which is going to have great potential in MSC preconditioning.

DFO and hypoxia-mimetic agents in optimized treatment conditions can improve MSC lifespan and maintain or increase their differentiation potential, migration, and immunomodulatory properties for successful engraftment in a hypoxia inducer concentration-dependent manner. The optimal culture conditions and pharmaceutical/chemical agent concentration should be optimized for priming stem cells to translate the results from *in vitro* effectiveness to *in vivo* conditions.

To summarize, preconditioning using DFO and other pharmacological/chemical hypoxia-mimetic agents positively affects MSC viability and other properties. They have not been studied so wildly as hypoxia but are believed to find application as pretreatment for many diseases considering their low cost and ease of use.

## Figures and Tables

**Table 1:** Effect of hypoxia on MSC surface markers.

Treatment conditions		Types of stem cells	The effect compared to normoxia (method of analysis)	Ref.
O_2_ concentration	Time/passage			
1%	2 d	hBM-MSC	No changes in positive (CD44 and TF) and negative markers (CD11b, CD19, CD34, CD45, and HLA-DR); decreased level of CD73, CD90, and CD105 (flow cytometry)	[57]
1%	10 d	hBM-MSC	No changes in positive (CD73, CD90, and TF) and negative markers (CD45, CD34, Cd11b, CD19, and HLA-DR); decreased level of CD105 and CD44 (flow cytometry)	[57]
1%	14 d	hBM-MSC	No changes in positive (CD73, CD90, CD105, CD106, CD 146, and MHC class I) and negative markers (CD45, CD34, and HLA-DR) (flow cytometry)	[58]
1%	21 d	hAD-MSC	No changes in positive (CD90 and CD105); increased level of negative markers (CD34, CD54, and CD 166) (flow cytometry)	[62]
2%	2 d	hBM-MSC	No changes in positive (CD73, CD44, CD90, and CD105) and negative markers (CD11b, CD19, CD45, CD34, and HLA-DR) (flow cytometry)	[57]
2%	12 d	hBM-MSC	No changes in positive (CD90, CD73, and CD105) and negative markers (CD31, CD34, CD45, and CD80) (flow cytometry)	[63]
2%	7 passages	hBM-MSC	Upregulation of *Oct4* and *REX-1* (RT-PCR)	[38]
2%	10 passages	hWJ-MSC	No changes in positive (CD73, CD105, CD90, CD44, CD10, CD29, and CD13) and negative markers (CD14, CD34, CD33, CD45, and HLA-DR) (flow cytometry); increased level of *DNMT3B*, *CRABP2*, *IL6ST*, *IFITM1*, *GRB7*, *IMP2*, *LIN28*, and *KIT* (RT-PCR)	[64]
2.5%^∗^	>72 h^∗^	hUCB-MSC	No changes in positive (CD44, CD73, CD90, and CD105) and negative markers (CD14, CD34, CD45, CD271, and HLA-DR) (flow cytometry); upregulation of *Oct4*, *NANOG* (RT-PCR)	[60]
2.5%^∗^	>72 h^∗^	hUCB-MSC	No changes in positive (CD73, CD44, CD105, and CD90) and negative markers (CD14, CD45, and CD106) (flow cytometry)	[65]
5%	2 d	hBM-MSC	No changes in positive (CD44, CD90, and CD73) and negative markers (CD11b, CD19, CD34, CD45, and HLA-DR); reduced level of CD105 (flow cytometry)	[57]
5%	14 d	hBM-MSC	No changes in positive (CD73, CD90, CD44, CD105, and STRO-1) and negative markers (CD34); reduced level of CD146 and CD45 (flow cytometry)	[65]
5%	Primary cells and passage 2	hBM-MSC	No changes in positive (CD29, CD73, CD90, CD44, CD105, and STRO-1) and negative markers (CD45 and CD34) (flow cytometry)	[66]

^∗^Hypoxic preconditioning in 2.5% O_2_ for 15 minutes, then reoxygenation at 21% O_2_ for 30 minutes, and again hypoxia preconditioning at 2.5% O_2_ for 3 days; h: human; d: day/days; UCB: umbilical cord blood.

**Table 2:** Effect of hypoxia on MSC viability, proliferation, and clonogenicity.

Treatment conditions		Types of stem cells	The effect compared to normoxia (methods of analysis)	Ref.
O_2_ concentration	Time/passage			
1%	2 d	hBM-MSC	Proliferation (DNA Quant-iT Picrogreen assay), clonogenicity (Giemsa staining), and metabolic activity (Vybrant assay) increased; *HIF-1α* downregulated (qRT-PCR), the proapoptotic genes: *BAX*, *BCL-2*, and *CASP-3* downregulated (qRT-PCR)	[57]
1%	2 d	rBM-MSC	The proliferation decreased (Trypan Blue staining, cell count)	[80]
1%	7 d	hBM-MSC	Proliferation significantly reduced (MTS proliferation assay)	[58]
1%	7 d	hBM-MSC	*HIF-1α* upregulated three-folds (qRT-PCR)	[58]
1%	9 d	hAD-MSC	Proliferation increased 1.7-folds (Trypan Blue staining, cell count)	[62]
1%	10 d	hBM-MSC	Proliferation (DNA Quant-iT Picrogreen assay) decreased, and metabolic activity increased (Vybrant assay), *HIF-1α* downregulated (qRT-PCR), the proapoptotic genes *BCL-2* and *CASP-3* downregulated, *BAX* upregulated (qRT-PCR)	[57]
1%	14 d	mBM-MSC	Viability (MTT viability assay) and proliferation (BrdU cell proliferation assay) increased, the main metabolic regulators like *Hk2* upregulated (sqRT-PCR), shift to anaerobic glycolysis, the *Slc16a3* (MCT-4) gene upregulated under prolonged hypoxia (qRT-PCT), the MCT-4 level increased under prolonged hypoxia (WB)	[68]
1%	14 d	rBM-MSC	Clonogenicity increased (crystal violet staining)	[80]
1%	21 d	hAD-MSC	Cell aging reduced, telomeres longer 1.5-folds (qPCR)	[62]
1%	21 d	hBM-MSC	A slowdown of cell cycle progression, accumulation in G1 phase under prolonged hypoxia (flow cytometry)	[58]
1-3%	16 h	hBM-MSC	Viability and proliferation (flow cytometry) maintained, Akt signaling pathway activated (WB)	[81]
1.5%	1 d	hBM-MSC hUCB-MSC	Proliferation increased (Trypan Blue staining, cell count) and the cell cycle faster progression (flow cytometry), HIF-1*α* increased (WB)	[82]
1.5%	3 d	hUC-MSC	Proliferation decreased (Trypan Blue staining, cell count), *LDHA, GLUT-1*, and *PDK-1* upregulated (RT-PCR), glutamate production decreased (HPLC), glucose consumption significantly increased (YSI 2700 analyzer)	[69]
2%	2 d	hBM-MSC	Proliferation (DNA Quant-iT Picrogreen assay), clonogenicity (Giemsa staining), and viability (flow cytometry) increased	[57]
2%	2 d	hWJ-MSC	Expression of the genes *HIF1-α*, *HIF-2α*, *Notch2*, and *JAGGED1* increased (RT-PCR)	[64]
2%	7 d	hBM-MSC	A high growth rate maintained even after confluency–multilayer formation (cell count, growth curve), *HIF-2α* upregulated (RT-PCR)	[38]
2%	7 d	hBM-MSC	Clonogenicity increased (crystal violet staining)	[63]
2%	12 d	hBM-MSC	Higher proliferation rate (Trypan Blue staining, cell count), the number of actively dividing cells significantly increased (PKH26 Red Fluorescent Cell Linker kit), the cellular division started earlier in the cell cycle (PKH26 staining, flow cytometry)	[63]
2%	20 d	hBM-MSC	Clonogenicity (colony count from microscopic images) and doubling time (cell count and growth curve) maintained, cellular senescence reduced (*β*-galactosidase staining, histochemistry)	[71]
2%	Passages 2-7	hBM-MSC	Higher cell number in each passage from 2 to 7 (Trypan Blue staining, cell count)	[38]
2%	10 passages	hWJ-MSC	Faster growth rates and higher total cell number yielded (cell area count, image analysis), normal karyotype maintained (Giemsa staining)	[64]
2%	64 d	hBM-MSC	Homogenous morphology of rapidly self-renewing cells maintained up to 52 d (microscopy analysis)	[83]
2.5%	3 d	hUC-MSC	Proliferation increased (cell counting under a microscope), HIF-1*α* increased (WB), *PDK-1, GLUT-1*, and *LDHA* upregulated (RT-PCR), glutamate production diminished (HPLC), glucose consumption significantly increased (YSI 2700 SELECT analyzer)	[69]
2.5% ∗	>3 d^∗^	hUCB-MSC	Cell viability (at 24 h and 2 d) increased (Trypan Blue staining, cell count, and MTT); proliferation (at 3 d) increased (Trypan Blue staining, cell count), CFU-F number *in vitro* significantly enhanced (Giemsa staining)	[60]
2.5% ∗	>3 d^∗^	hUCB-MSCs	Cell metabolic activity (MTT), CFU-F number (Giemsa staining), and proliferation (at 2 and 3 d) (Trypan Blue staining, cell count) increased, doubling time reduced (at 2 and 3 d) (Trypan Blue staining, cell count), cell death inhibited (at 2 and 3 d) (microscope analysis)	[84]
3%	~100 d Passage 1	hBM-MSC	Proliferative lifespan with additional 10 PD improved (flow cytometry), transcription of hypoxia-related the genes encoding *VHL*, *HIF-1*, *PH-4*, *HYOU1*, *HIF1AN*, *HIG*, and *HIG* unaltered (qPCR)	[33]
3%	Over 25 passages	hBM-MSC	Cell growth improved (Trypan Blue staining, cell count), population doublings increased (Trypan Blue staining, cell count), oxidative stress reactions (DHE, flow cytometry) and nuclear alterations such as damage of DNA, telomere shortening, and chromosomal abnormalities (DAPI, Q-FISH, Breast Aneusomy Multicolor Probe kit) limited, glycolysis increased (OCR/ECAR, F96 Flux analyzer)	[32]
5%	2 d	hBM-MSC	Proliferation rate lowered (DNA Quant-iT Picrogreen assay), clonogenicity (Giemsa staining), and metabolic activity elevated (Vybrant assay)	[57]
5%	3 d	hUC-MSC	Proliferation increased (Trypan Blue staining, cell count), *LDHA*, *PDK-1*, and *GLUT-1* upregulation (RT-PCR)	[69]
5%	4 d	rBM-MSC	Proliferation rate increased (flow cytometry)	[85]
5%	4 d	hBM-MSC	Clonogenicity (crystal violet staining), proliferation (EDU Proliferation kit), and metabolic activity (Alamar Blue staining) increased	[65]
5%	14 d	hBM-MSC	Clonogenicity decreased at primary cells and the passage 1 but increased at the passages 2 and 3 (crystal violet staining)	[66]
5%	20 d	hBM-MSC	Colony formation significantly reduced (colony count from microscopic images), doubling time maintained (Trypan Blue staining, cell count, growth curve), cellular senescence reduced (*β*-galactosidase staining, histochemistry, blue stained cell count)	[71]
5%	Passage 1-10	hBM-MSC	The number of population doublings increased (Trypan Blue staining, cell count), cellular senescence reduced (*β*-galactosidase staining, histochemistry, blue stained cell count)	[72]

^∗^Hypoxic preconditioning in 2.5% O_2_ for 15 minutes, then reoxygenation at 21% O_2_ for 30 minutes, and again hypoxia preconditioning at 2.5% O_2_ for 3 days; d: day/days; h: human; m: mouse; r: rat; PD: population doublings; MTT: 3-(4,5-dimethylthiazol-2-yl)-2,5-diphenyltetrazolium bromide; BrdU: 5-bromo tetrazolium inner salt-20-deoxyuridine; MTS: tetrazolium inner salt; WB: Western Blotting.

**Table 3:** Effect of pharmaceutically and chemically derived hypoxia on MSC viability, proliferation, and clonogenicity.

Treatment conditions		Stem cell type	The effect compared to normoxia (methods of analysis)	Ref.
An agent/concentration	Time			
DFO/0.1 *μ*M	53 d	hBM-MSC	Proliferation increased (Incucyte HD Imaging system)	[61]
DFO/10 *μ*M	2 d	hBM-MSC	The genes related to glycolysis (*HK2*, *PDK-1*, *BNIP3*, *LDHA*), viability, and survival upregulated (microarray analysis)	[61]
DFO/10 *μ*M	53 d	hBM-MSC	Proliferation inhibited at concentrations of 10 *μ*M and higher (Incucyte HD Imaging system)	[61]
DFO/50 *μ*M	12 h	hBM-MSC	Proliferation as effective as for 2 d in 3 *μ*M DFO (Incucyte HD Imaging system), HIF-1*α* upregulated (microarray analysis)	[99]
DFO/50 *μ*M	1-3 d	rBM-MSC	Viability increased (MTT)	[100]
DFO/50-500 *μ*M	1 d	hAD-MSC	Viability unchanged (CellTiter 96 Aqueous kit)	[102]
DFO/100 *μ*M	12 h	rBM-MSC	*HIF-1α* increased (qRT-PCR)	[100]
DFO/100 *μ*M	1 d	rBM-MSC	HIF-1*α* increased (WB)	[100]
DFO/100 *μ*M	1-3 d	rBM-MSC	Viability increased (MTT)	[100]
DFO/100 *μ*M	2 d	hWJ-MSC	HIF-1*α* increased (WB)	[115]
DFO/120 *μ*M	2 d	hUC-MSC	Cell viability was DPO concentration-dependent, cell viability decreased above 120 *μ*M DFO (MTT)	[97]
CoCl_2_/50-300 *μ*M	1 d	hAD-MSC	Viability increased (MTT)	[102]
CoCl_2_/100 *μ*M	1-2 d	hDP-MSC hUC-MSC hAD-MSC	Viability increased (MTT)	[53]
CoCl_2_/100 *μ*M	2 d	hDP-MSC hUC-MSC hAD-MSC	HIF-1*α* increased in DP- and UC-MSC and maintained in AD-MSC (WB)	[53]
CoCl_2_/100 *μ*M	2 d	hUC-MSC	Viability decreased above 100 *μ*M CoCl_2_ (MTT)	[97]
CoCl_2_/100 *μ*M	6 d	Coculture hBM-MSC HUVEC	The higher proliferation of hBM-MSC in coculture (crystal violet staining), reduced viability of hBM-MSC	[52]
CoCl_2_/0.5 mM	1 d	hAD-MSC	Reduced viability (MTT)	[102]
DMOG/100 *μ*M+SD	1 d	rBM-MSC	Proliferation maintained (Trypan Blue staining, cell count), PI3K/Akt signaling activated (WB), HIF-1*α* increased (WB)	[103]
DMOG/0.5 mM + SD	1 d	rBM-MSC	Proliferation maintained (Trypan Blue staining, cell count), PI3K/Akt signaling activated (WB), HIF-1*α* increased (WB)	[103]
DMOG/0.5 mM+ 1%O_2_	2 d	rBM-MSC	HIF-1*α* increased (WB)	[80]
DMOG/0.5 mM	6 d	Coculture hBM-MSC HUVEC	The higher proliferation of hBM-MSC in coculture (crystal violet staining), increased viability of hBM-MSC	[52]
DMOG/1 mM	1 d	rBM-MSC	Viability increased in vitro (Hoechst 33342 staining), HIF-1*α* increased (WB), glucose transporter 1 increased (WB), the pAKT level increased (WB), increase survival of MSC after transplantation into ischemic heart (a rat model) (TUNEL assay), time-dependent protective effect against cell death in vitro (Trypan Blue staining, cell count)	[92]
DMOG/1 mM + SD	1 d	rBM-MSC	Proliferation maintained (Trypan Blue staining, cell count), PI3K/Akt signaling activated (WB), HIF-1*α* increased (WB)	[103]
DMOG/5 mM + SD	1 d	rBM-MSC	Proliferation decreased (Trypan Blue staining, cell count)	[103]
ISO/2%	4 h	hBM-MSC	Cell metabolic activity increased after 4 h, significantly reduced after 6 h at ISO concentrations above 2% (MTT), HIF-1*α* increased (WB), the PI3K/Akt signaling activated (WB), the percentage of apoptotic cells significantly reduced after treatment with 1-2% ISO for 6 h (flow cytometry)	[98]
DNP/0.25 mM^∗^	20 min^∗^	Coculture rBM-MSC CM	The viability significantly increased (PKH26, flow cytometry)	[94]

^∗^20 minutes of treatment with 0.25 mM and then reoxidation either 2 or 24 hours in 21% O_2_; d: day/days; h: human; m: mouse; r: rat; SD: serum deprivation; CM: cardiomyocytes; HUVEC: human umbilical vein endothelial cells.

**Table 4:** Effect of hypoxia pre/treatment on MSC differentiation.

Treatment conditions		Type of stem cells	The effect compared to normoxia (methods of analysis)	Ref.
O_2_ concentration	Time/passage			
1%	Pretreatment for 2 d	rBM-MSC	Osteogenesis increased (ALP activity, 7 d; Alizarin Red S, 21 d)	[80]
1%	Pretreatment for 3 d	hBM-MSC	Osteogenic potential of MSC maintained, for high-density spheroid osteogenic potential enhanced; increase in the ALP activity related to the spheroid cell density (ALP staining after 14 d), osteocalcin level maintained (ELISA)	[89]
1%	Pretreatment for 14 d	hBM-MSC	Adipogenic differentiation impaired (Oil Red staining, 14-20 d), osteogenic potential reduced (Alizarin Red staining, 21 d, calcium precipitates detected)	[58]
1%	Pretreatment for 21 d	hAD-MSC	Chondrogenic potential increased (Alcian Blue staining, 21 d); high expression of the *SOX9* and *COL2A1* genes (RT-PCR), osteogenic potential slightly reduced (Von Kossa staining, 21 d, manual counting of calcified areas); lower expression of *ALP* and unchanged *OPN* (RT-PCR), adipogenic potential slightly reduced (Oil Red staining, 21 d); lower expression of the *ADPN* and *LPL* genes (RT-PCR)	[62]
1%	Treatment for 9 d	Coculture hBM-MSC HUVEC	Osteogenic potential maintained (Alizarin Red stain, 9 d), expression of the osteogenic *RUNX2* and *ALP* genes retained and upregulation *COLIA1* (qRT-PCR)	[52]
1.5%	Pretreatment for 1 d	BM-MSC UCB-MSC	Osteogenic potential (Von Kossa staining, 14 d) and expression of the *RUNX2* gene retained (RT-PCR), adipogenic differentiation potential retained (Oil Red staining, 21Coll type IId) and its corresponding marker gene *PPAR-α* (RT-PCR), chondrogenesis (Coll type II detection, 21d), and expression of the *SOX9* gene increased (RT-PCR)	[82]
2%	Pretreatment for 7 d	hAD-MSC	Osteogenesis enhanced (Von Kossa staining, 22Coll type II d), adipogenesis enhanced (Oil Red staining, 21Coll type II d)	[114]
2%	Pretreatment for 8 d	mAD-MSC	Early chondrogenesis increased (Alcian Blue staining, sGAGs assay kit, 6 d), osteogenesis after 7 d maintained (ALP activity, colorimetric assay), after 21 d—decreased (Alizarin Red staining)	[116]
2%	Pretreatment at passage 2 and/or treatment for 21 d	hBM-MSC	Osteogenic potential reduced (Alizarin Red staining, 21 d), osteogenic potential reduced due to hypoxia pretreatment in cells grown in normoxia and hypoxia conditions	[71]
2%	Treatment for 14 d	hBM-MSC	Osteogenic potential maintained (Von Kossa staining and ALP activity, 14 d), adipogenic potential maintained (Oil Red staining, 14 d)	[63]
2%	Treatment for 14 d	hBM-MSC	Osteogenic potential preserved (Von Kossa staining, 14 d), the above capacities preserved up to the seventh passage	[38]
2%	Treatment for 14-17 d	hWJ-MSC	Chondrogenic potential maintained (Alcian Blue staining, 14-17 d)	[64]
2%	Treatment for 18 days	hWJ-MSC	Adipogenic potential increased (Oil Red staining, 18 d)	[63]
2%	Treatment for 21 days	hBM-MSC	Adipogenic potential preserved (Nile Red staining, 21 d) up to the seventh passage	[38]
2%	Treatment for 21 days	hWJ-MSC	Osteogenic potential increased (Von Kossa staining, 21 d)	[64]
2.5%^∗^	Pretreatment for more than 3 days^∗^	hUC-MSC	Osteogenic (Alizarin Red staining, 21 d), adipogenic (Oil Red staining, 21 d), and chondrogenic (Alcian Blue staining, 21 d) potential preserved	[84]
3%	Pretreatment for 7-10 d and/or treatment for 21 d	hBM-MSC	Both hypoxia pretreatment and hypoxic treatment during differentiation preserve osteogenic (Alizarin Red staining, 21 d) and adipogenic (Oil Red staining, 21 d) potential for primary cells only, diminished adipogenesis and inhibited osteogenesis with increase in the passage number from 1 to 4, the genes *ALPL*, *IBSP*, *FABP4*, and *LPL* downregulated (qRT-PCR) with increase in the passage number from 1 to 4, osteogenic differentiation (passage 1) stimulated upon transition from 3 to 20% O_2_	[33]
1-3%	Pretreatment for 16 h	hBM-MSC	Osteogenic (Alizarin Red staining), adipogenic (Oil Red staining), and chondrogenic (Alcian Blue staining) differential potential maintained	[81]
1-5%	Treatment for 21 d	hBM-MSC	Osteogenic differentiation reduced (Alizarin Red assay, 7, 14, and 21 d), ALP and OPN expressed at low levels below 5% O_2_ (WB)	[125]
5%	Pretreatment at passages 2-4 and/or treatment for 21 d	hWJ-MSC	Osteogenesis (Von Kossa staining, 21 d) and cartilage differentiation (Masson's trichrome staining, 21 d) maintained at the same level, hypoxic/normoxic pretreatment and treatment did not affect MSC differentiation potential	[126]
5%	Pretreatment up to passage 2 and/or treatment for 28 d	hBM-MSC	Osteogenic potential (Alizarin Red staining, 28 d), and the expression of the *ALPL* and *RUNX2* genes increased (RT-PCR), adipogenic potential (Oil Red staining, 28 d) increased; the expression level of the *LPL* and *PPARγ* genes maintained (RT-PCR), both hypoxia pretreatment and hypoxic treatment increased differentiation potential	[66]
5%	Treatment for 14 d	hBM-MSC	Osteogenic potential (ALP activity, 14 d) and the *BGLAP*, *RUNX2*, and *COLL2* gene expression increased (RT-PCR)	[65]
5%	Treatment for 21 d	hBM-MSC	Adipogenesis reduced (Oil Red staining, 21 d)	[65]
5%	Treatment for 28 d	hBM-MSC	Chondrogenesis reduced (Alcian Blue staining, 28 d)	[65]
8%	Treatment for 8 d	mBM-MSC	Adipogenesis increased after 8 d (Sudan Black staining)	[123]
8%	Treatment for 14 d	mBM-MSC	*Oct4* inhibited (qRT-PCR)	[123]

^∗^Hypoxic preconditioning in 2.5% O_2_ for 15 minutes, then reoxygenation at 21% O_2_ for 30 minutes, and again hypoxia preconditioning at 2.5% O_2_ for 3 days; d: day/days; h: human; m: mouse; r: rat.

**Table 5:** Effect of pharmaceutically and chemically derived hypoxia pretreatment on MSC differentiation.

Treatment conditions		Stem cell type	The effect compared to normoxia (methods of analysis)	Ref
An agent/concentration	Time			
DFO/3 *μ*M	Treatment for 14 d	hBM-MSC	Osteogenic (Alizarin Red staining, 14 d) and adipogenic (Oil Red staining, 14 d) potential decreased	[61]
DFO/15 *μ*M	Treatment for 7 d	hBM-MSC	ALP increased (WB, 7 d)	[120]
DFO/15 *μ*M	Treatment for 21 d	hBM-MSC	Osteogenic potential increased (Alizarin Red staining, 21 d), *ALP*, *RUNX2*, and *OC* upregulated (qRT-PCR), osteogenesis through *β*-catenin signaling increased (WB)	[120]
DFO/50 *μ*M	Treatment for 21 d	hBM-MSC	Chondrogenesis (Alcian Blue, 21 d) and *SOX9* expression (RT-PCR, 7 d) slightly increased	[124]
DFO/120 *μ*M	Treatment for 8 d	mBM-MSC	Adipogenic potential preserved (Sudan Black staining, 8 d), *Oct4* expression maintained (qRT-PCR)	[123]
DFO/120 *μ*M	Treatment for 21 d	hUC-MSC	Osteogenic (Von Kossa staining, 21 d), and adipogenic (Oil Red staining, 21 d) potential preserved	[97]
CoCl_2_/100 *μ*M	Pretreatment for 1-2 d	mC3H/10T1/2-MSC	Osteogenesis (Alizarin Red staining, 18 d) and expression of the *Col I*, *Bglap*, and *Alp* genes (RT-PCR, 10 d) enhanced, adipogenesis (Oil Red staining, 5 d) and expression of the *Ap2*, *C/ebpα*, and *Pparγ* genes (RT-PCR, 5 d) reduced, chondrogenesis (Alcian Blue staining, 14 d) and expression of *SOX9*, *Col II*, and *ACAN* genes (RT-PCR, 10 d) enhanced	[101]
CoCl_2_/100 *μ*M	Pretreatment for 2 d	hDP-MSC hUC-MSC hAD-MSC	Chondrogenesis (Alcian Blue, 28 d) in AD- and UC-MSC increased, in DP-MSC—not detected, cell-source dependent changes of the expression of the following genes: *COL2A1*, *ACAN*, *SOX9*, and *VCAN* (RT-PCR): DP-MSC–*SOX9* (after 7 d up, and after 14, 21, and 28 d downregulated), *VCAN* (after 7, 14, 21, and 28 d upregulated), *COL2A1* and *ACAN* not detected UC-MSC–*SOX9* (after 7, 28 d down, and after 14 and 21 d upregulated), *COL2A1*, *ACAN*, and *VCAN* (after 7,14, 21, and 28 d—upregulated) AD-MSC–*SOX9* (after 7 d up-, and after 14, 21, and 28 d downregulated), *COL2A1* (after 7,14, 21, and 28 d upregulated), and *ACAN* upregulated only after 14 d, *VCAN* (after 7,14, 21, and 28 d downregulated)	[53]
CoCl_2_/100 *μ*M	Treatment for 8 d	mBM-MSC	Adipogenic potential preserved (Sudan Black staining, 8 d), *Oct4* expression maintained (qRT-PCR)	[123]
CoCl_2_/100 *μ*M	Treatment for 9 d	Coculture hBM-MSC HUVEC	Osteogenic potential retained (Alizarin Red, nine days), expression of *RUNX2*, *ALP*, *and COLIA1* maintained (qRT-PCR)	[52]
CoCl_2_/100 *μ*M	Treatment for 21 d	hUC-MSC	Osteogenic (Von Kossa staining, 21 d), and adipogenic (Oil Red staining, 21 d) potential preserved	[97]
CoCl_2_/100 *μ*M	Treatment for 21 d	hBM-MSC	Chondrogenesis (Alcian Blue, 21 d) and *SOX9* expression (RT-PCR 7d) maintained	[124]
DMOG/200 *μ*M	Treatment for 21 d	hBM-MSC	Chondrogenesis (Alcian Blue, 21 d) and *SOX9* and *RUNX2* expression (RT-PCR, 7 d) increased	[124]
DMOG/500 *μ*M	Pretreatment for 2 d	rBM-MSC	Osteogenesis (Alizarin Red S, 21 d) and ALP activity (7 d) increased	[80]
DMOG/500 *μ*M +1%O_2_	Pretreatment for 2 d	rBM-MSC	Osteogenesis (Alizarin Red S, 21 d) and ALP activity (7 d) increased	[80]
DMOG/500 *μ*M	Treatment for 9 d	Coculture hBM-MSC HUVEC	Osteogenic potential retained (Alizarin Red, 9 d), expression of *RUNX2* maintained (qRT-PCR), expression of *ALP* and *COLIA1* increased (qRT-PCR)	[52]

d: day/days; h: human; m: mouse; r: rat.

**Table 6:** Effect of hypoxia on MSC engraftment, migration, and secretion profile.

Treatment conditions		Stem cell type	The effect compared to normoxia (methods of analysis)	Ref.
O_2_ concentration	Time/passage			
1%	1 d	hBM-MSC	*CX3CR1*and *CXCR4* upregulated (qRT-PCR)	[172]
1%	2 d	hBM-MSC	VEGF secretion in spheroids increased (ELISA) on a rat model, collagen deposition (Masson's trichrome stain) enhanced, vascularization and bone formation promoted (high-resolution radiographs), and healing after transplantation of primed MSC spheroids improved compared to transplantation of individual cells	[89]
1%	2 d	hBM-MSC	*VEGF* and *NANOG* upregulated (qRT-PCR)	[57]
1%	2 d 2 d	rBM-MSC	*VEGF* upregulated (RT-PCR), VEGF increased (WB, ELISA)	[80]
1%∗	>2 d^∗^	mBM-MSC	*Cxcr4 downregulated (qRT-PCR)*	[143]
1%	10 d	hBM-MSC	*VEGF* and *NANOG* upregulated (qRT-PCR)	[57]
1%	14 d	mBM-MSC	On a myocardium infarction (MI) mouse model, cardiomyocyte survival reduced due to MCT-4 (WB) increase, and fibrosis in cardiac tissue initiated	[68]
1-3%	16 h	hBM-MSC	Migration potential increased (scratch test)	[81]
2%	Up to 7 passages	hBM-MSC	ECM secretion enhanced (fibronectin and collagen type II fluorescent staining, CLSM), expression of connexin-43 increased (fluorescent staining, CLSM)	[38]
2.5%^∗∗^	>3 d^∗∗^	hUCB-MSC	Migration potential increased	[60]
5%^∗∗∗^	>8 h^∗∗∗^	mBM-MSC	CXCR4, MMP 9, and 14 increased (WB), after MI treatment on the rat model the left ventricular (LV) fibrosis reduced, improved LV function	[152]
5%	4 d	hBM-MSC	VEGF increased (ELISA)	[65]
5%	10 d	hBM-MSC	*MMP7-16* and *TIMP1-3* upregulated (qRT-PCR)	[65]

^∗^Hypoxic pretreatment 4-48 hours at 1% O_2_ and then reoxidation 8 hours at 21% O_2_; ^∗∗^hypoxic preconditioning in 2.5% O_2_ for 15 minutes, then reoxygenation at 21% O_2_ for 30 minutes, and again hypoxia preconditioning at 2.5% O_2_ for 3 days; ^∗∗∗^hypoxic pretreatment 8 hours at 5% O_2_ and then 30 minutes of reoxidation at 21% O_2_; h: human; m: mouse; r: rat.

**Table 7:** Effect of pharmaceutically and chemically derived hypoxia on MSC engraftment, migration, and secretion profile.

Treatment conditions		Stem cell type	The effect compared to normoxia (methods of analysis)	Ref
An agent/concentration	Time			
DFO/10 *μ*M	2 days	hBM-MSC	*VEGF* upregulated (RT-PCR)	[61]
DFO/50-300 *μ*M	1 day	hAD-MSC	*VEGF* increased, the higher DFO concentration induced the higher *VEGF* expression (qPCR)	[102]
DFO/60-600 *μ*M	20 h	hBM-MSC	*CX3CR1*and *CXCR4* upregulated (RT-PCR), CX3CR1 and CXCR4 increased (WB)	[172]
DFO/100 *μ*M	1-3 days	rBM-MSC	*Cxcr4* upregulated (RT-PCR), homing capacities in a NIHL rat model enhanced via PI3K/AKT signal transduction pathway (WB)	[100]
DFO/100 *μ*M	2 days	hWJ-MSC	*VEGF* upregulated (qRT-PCR), mobilization and homing capacities increased	[115]
DFO/150 *μ*M	1 day	hAD-MSC	VEGF increased (ELISA)	[102]
CoCl_2_/50-300 *μ*M	1 day	hAD-MSC	*VEGF* increased, the higher CoCl_2_ concentration the higher *VEGF* expression in the range of 50-150 *μ*M, at 300 *μ*M slightly dropped compared to *VEGF* expression at 150 *μ*M (qPCR)	[102]
DMOG/500 *μ*M	1 day	hBM-MSC	VEGF increased (WB), angiogenesis increased (tube formation test in the Matrigel), engraftment ability improved, cardiac function improved (left ventricular ejection fraction evaluation), rat model of MI	[92]
DMOG/500 *μ*M +1%O_2_	2 days	rBM-MSC	*VEGF* upregulated (RT-PCR), VEGF increased (WB, ELISA), angiogenic capability increased in vitro and *in vivo* (tube formation test, Matrigel, rat bone defect model)	[80]
DNP/0.25 mM	20 min	rBM-MSC	The cardiomyogenic genes (*Anp*, *Gata-4*, *Nkx2.5*, *Vegf*, and *Con43*) upregulated (RT-PCR); improvement in cardiac function and significant reduction in scar formation in the rat model of MI	[159]
DNP^∗^/0.25 mM	20 min^∗^	Coculture rBM-MSC CM	*Igf*, *Hgf*, *Vegf*, *Il-7*, *and Il-7r* upregulated (RT-PCR)	[94]
ISO/2%	4 h	hBM-MSC	CXCR4 increased (WB), cell migration increased (hematoxylin and eosin staining, cell count) on a rat stroke model, engraftment and recovery improved	[98]

^∗^20 minutes of treatment with 0.25 mM and then reoxidation either 2 hours or 1 day in 21% O_2_. h: human; r: rat.

## Abbreviations

AD: Adipose-derived
*α*-KG: Alpha ketoglutarate
*ALP*: *Alkaline phosphatase*
ANP: Atrial natriuretic peptide
aP2: Adaptor *protein* 2
BAX: BCL-2-associated X
BCL-2: B-cell lymphoma 2
bFGF: Basic fibroblast growth factor
BGLAP: Bone gamma-carboxyglutamic acid-containing protein (osteocalcin)
BM: Bone marrow
CASP-3: Caspase 3
CD: Crohn's disease
C/EBP*α*: CCAAT enhancer binding proteins
CBP/p30: CREB-binding *protein*/p300 protein
CCL25: C–C motif chemokine ligand 25
CMs: Cardiomyocytes
CoCl_2_: Cobalt chloride
Coll2a1: Collagen alpha-1(II) chain
Cx43: Connexin-43
CX3CL1: C-X3-C motif chemokine ligand 1
CX3CR1: C-X3-C motif chemokine receptor 1
CXCR4: C-X-C motif chemokine receptor 4
CXCR7: C-X-C chemokine receptor type 7
DFO: Deferoxamine
DNP: 2,4-Dinitrophenol
DM: Diabetes mellitus
DMOG: Dimethyloxaloylglycine
ESC: Embryonic stem cell
FABP4: Fatty acid-binding protein 4
*Gata-4*: *Gata*-binding *protein 4*
GLUT-1: Cellular glucose transporter-1
G6PD: Glucose-6-phosphate dehydrogenase
GVHD: Graft-versus-host disease
hESC: Human embryonic stem cells
HIF-1*α*: Hypoxia-inducible factor 1 alpha
hiPSC: Human-induced pluripotent stem cells
HK2: Hexokinase 2
HLA-DR: Human leukocyte antigen-DR isotype
HMGB1: High mobility group box protein 1
HREs: Hypoxia response elements
HUVEC: Human umbilical vein endothelial cells
IBSP: Integrin-binding sialoprotein
LDHA: Lactate dehydrogenase A
LPL: Lipoprotein lipase
mC3H/10T1/2: Murine embryonic mesenchymal cell line
MMP-9: Matrix metalloproteinase 9
MS: Multiple sclerosis
MSC: Mesenchymal stem cell
NANOG: Homeobox transcription factor
Nkx2.5: NKX2 homeobox *5*
*Notch*: Translocation-associated (Drosophila), a *signaling pathway*
ISCT: International Society for Cellular Therapy
ISO: Isoflurane
Oct4: Octamer-binding transcriptional factor 4
2-OG: 2-Oxoglutarate
P53: Cellular tumor antigen
PDK-1: 3-Phosphoinositide-dependent protein kinase 1
PHD: Oxygen-dependent prolyl hydroxylases
PGF: Placental growth factor
PI3K: Phosphoinositide 3-kinases
PRPP: Phosphoribosyl pyrophosphate
pVHL: von Hippel–Lindau protein
REX-1: Reduced expression protein-1
ROS: Reactive oxygen species
RUNX2: Runt-related transcription factor 2
SOX2: SRY-box transcription factor 2
SOX9: SRY-box transcription factor 9
SSEA-3: Stage-specific mouse embryonic antigen-3
SSEA-4: Stage-specific mouse embryonic antigen-4
TCA: Tricarboxylic acid cycle
TIMPs: Tissue inhibitor of metalloproteinases
Tra-1-60: T cell receptor alpha locus 1-60
UC: Umbilical cord
UCB: Umbilical cord blood
WJ: Wharton's jelly
VEGF: Vascular endothelial growth factor
VEFG-D: Vascular endothelial growth factor-D
VCAN: Versican core protein
VWF: von Willebrand factor.
